# It Pays to Be Pushy: Intracohort Interference Competition between Two Reef Fishes

**DOI:** 10.1371/journal.pone.0042590

**Published:** 2012-08-10

**Authors:** Mark I. McCormick, Christine J. Weaver

**Affiliations:** ARC Centre of Excellence for Coral Reef Studies, and School of Marine and Tropical Biology, James Cook University, Townsville, Queensland, Australia; University of Hamburg, Germany

## Abstract

Competition is often most intense between similar sized organisms that have similar ecological requirements. Many coral reef fish species settle preferentially to live coral at the end of their larval phase where they interact with other species that recruited to the same habitat patch at a similar time. Mortality is high and usually selective and individuals must compete for low risk space. This study examined the competitive interactions between two species of juvenile damselfish and the extent to which interactions that occurred within a recruitment cohort established the disjunct distribution patterns that were displayed in later life stages. Censuses and field experiments with juveniles found that one species, the ambon damsel, was dominant immediately after settlement and pushed the subordinate species higher up the reef and further from shelter. Presence of a competitor resulted in reduced growth for both species. Juvenile size was the best predictor of competitive success and outweighed the effects of short term prior residency. Size at settlement also dramatically influenced survival, with slightly larger individuals displaying higher aggression, pushing the subordinate species into higher risk habitats. While subordinates had higher feeding rates, they also sustained higher mortality. The study highlights the importance of interaction dynamics between species within a recruitment cohort to patterns of growth and distribution of species within communities.

## Introduction

Interspecific competition is a key process affecting resource acquisition, growth and survival of organisms within and among habitats. Manipulative experiments have shown that competition between adults, whether exploitative or interference, leads to exclusion of inferior resource competitors from some habitats when intense [1]–[4]. Dominant competitors may reduce growth of the subordinate individuals directly or indirectly enhancing mortality of inferior competitors. Adults of one species may also have an influence on the growth or survival of younger stages of a competing species [5]–[10]. Despite its demonstrated importance for established life stages, the effects of interspecific competition within cohorts of young-of-the-year individuals has seldom been examined (see [11] for an exception). Young-of-the-year are those individuals most vulnerable to resource restriction. Limited storage reserves mean that small changes in key resources can lead to marked reductions in growth and survival, which will carry-over to the numbers of individuals in the next life stage and species distributions patterns in general [12].

Young-of-the-year are particularly vulnerable when they are from taxa with complex life cycles, such as many insects, amphibians and fishes [13], [14]. Established populations of these organisms are replenished by juveniles entering an environment that is often different from their natal environment with respect to the habitat characteristics and species composition. Periodic spawning synchronized by environmental rhythms, and typically low variability in larval duration, means that newly metamorphosed individuals enter habitat patches in pulses that include a variety of similar species (e.g. [15]). Newly metamorphosed individuals must immediately interact with individuals of their own and other species for space and vital resources. The outcome of these interactions and the fate of individuals may be closely tied to their size at settlement since organisms such as fishes and amphibians differ markedly in their size at settlement among ecologically similar species (e.g. [16], [17]), within species (e.g. [18], [19]) and within recruitment pulses of an individual species (e.g. [20], [21]). This means that organisms settling to the same habitat may compete directly with larger, smaller or similar sized individuals.

Individuals and species may also enter a habitat patch at slightly different times. Priority effects have been shown to dramatically influence the outcome of competitive interactions, with the ‘home advantage’ often reversing the outcome of competitive interactions between species all else being equal (e.g. [22], [23]). Previous experiments have shown that the timing of entry to a habitat in relation to a competitor is a key contributor to whether an individual survives in a unit of habitat. Such intracohort priority effects have been found in a diversity of organisms including bacteria [24], zooplankton [25], insects [26], amphibians [27] and fishes [23]. Knowledge of such temporal effects is critical to estimates of the strength of competition and interpretation of the importance of key processes influencing community dynamics.

Tropical marine fishes are ideal organisms in which to study the influence of intracohort interspecific competition on behaviour, growth, distribution and survival. Many species use live hard coral as a preferred settlement habitat. Jones et al. [28] estimated 65% of fishes within a diverse tropical fish assemblage use live coral as a nursery habitat. Tropical fishes also settle at the end of their larval phase in pulses associated with the lunar phases [15], which results in a diversity of species arriving at a coral habitat patch at approximately the same time. Thus habitat preference and recruitment timing bring about a situation that promotes both intra- and inter-specific competition at a life stage when the small fish are highly vulnerable. Mortality during the first week after settlement is typically extremely high and selective [29], [30] and at least part of this mortality is mediated through behavioural interactions within and among species [23], [31]–[33]. While there was substantial debate in the 1980’s about the importance of competition at early life stages in fishes (reviewed in [34], [35]), few studies have explored the importance of the role of competition in influencing the distribution and survival of individuals at this important life history boundary.

The goal of the present study was to examine the extent to which competition among juveniles influenced the distribution, growth and survival of two congeneric species of planktivorous damselfishes *Pomacentrus amboinensis* and *P. moluccensis*. Experimental field manipulations examined the behavioural mechanisms underlying interference competition between species and its impact on growth and survival. A second series of experiments examined the importance of size and prior residency in influencing the outcomes of behavioural interactions. We predicted that: 1) dominant individuals should use more of the preferred habitat than subordinates, 2) if competition was strong, it would influence growth and survival; 3) size would have a major influence on dominance, and would be more important than prior residency; 4) dominance would be linked to survival through modified space use mediated by behavioural interactions.

## Materials and Methods

### Study Species and Location

The ambon damselfish *Pomacentrus amboinensis* and lemon damsel *P. moluccensis* are common site attached species of damselfish (family Pomacentridae) found throughout the Indo-Pacific on shallow reef habitats at the interface between the live coral and rubble reef edge (Fig. S1). Both species have a similar larval duration after a demersal egg phase and settle at similar sizes (*P. amboinensis* 17.8 d, 11.2 mm SL; *P. moluccensis* 19.4 d, 10.7 mm SL; [17]). Metamorphosis is concomitant with settlement and in these species involves a major change in pigmentation (transparent to coloured) that occurs within hours, but involves little obvious change in shape [36]. However, settlement does involve major changes in physiology [37] and it is likely that marked changes also occur in the sensory systems [38]. A laboratory-based habitat selection experiment has previously shown that both species preferentially settle to healthy live coral [39]. Both species settle naturally to patches of mixed live and dead coral. Both are also planktivores as juveniles and eat a similar array of prey items (Text S1). A tagging study of 295 newly settled *P. amboinensis* on the continuous reef edge found that fish moved little over the first 3 months after settlement (mean = 0.63 m [40]). It is likely that *P. moluccensis* has a similar degree of site attachment (pers. obs.).

Research on newly settled *P. amboinensis* has shown that fish enter the reef with high variability in their behavioural traits (e.g. boldness, aggression) and these traits are displayed in a manner that is consistent on small time scales of hours to days ([41], [42], Mero, Meekan and McCormick unpublished data]. Establishment of dominance hierarchies occurs within minutes of settlement within the species, which can rapidly lead to the eviction of subordinates from small habitat patches [31]. Because of the rapid establishment of territories and the high juvenile mortality, it was decided that 60 min was an ecologically relevant time to use for the establishment of residents in the priority experiments for the present study.

The present datasets were collected at Lizard Island (14° 40′S 145° 28′ E) on the northern Great Barrier Reef, Australia, between October 2007 and March 2010. Both newly metamorphosed juveniles and recently settled juveniles from the reef were used for field experiments. Light traps (see [43] for design; small trap) were used to collect both fish species at the end of their larval phase prior to their settlement to the reef. These newly metamorphosed fish were separated by species and placed into 60 L aquaria with aerated flowing seawater. Fish were kept for 24 h and fed newly hatched *Artemia* sp. twice per day *ad libitum* to allow recovery from (or acclimation to) the stress of capture, prior to use in experiments. Juveniles were collected from a shallow fringing reef at the back of Lizard Island using a solution of dilute clove oil and hand nets. All fishes used in the experiments were placed into a small clip-seal bag with a small amount of aerated seawater and measured with calipers (±0.1 mm) and then transferred into individually labeled 1 L clip-seal bags for transport. To reduce transport and handling stress, fish in bags were transported to the field site in a 30 L bin of seawater (to reduce temperature fluctuations) under subdued light conditions.

### Habitat Use

To quantify the spatial organization of *P. amboinensis* and *P. moluccensis* juveniles in the field the small scale spatial pattern of juveniles on the leeward reef edge at the sand-coral interface was recorded. This was a common habitat for the two species and a diverse reef fish assemblage was also present within the area. Areas of reef were chosen where juveniles of both species were present within 1.5 m of one another. To enable quantification, habitat chosen for sampling was standardized: areas of the reef edge where the distance between the sand and top of the reef was approximately 1.5 m (range: 1.2–1.8 m), with coral rubble near the sand, grading into live coral (mostly the bushy hard coral *Pocillopora damicornis*) at the reef top. Within the constraint of this designation, sampling areas were chosen randomly. The first juvenile of either target species was placed into one of three categories of juveniles based on their size and colouration [42]: (1) recent recruits (within the last week, ∼10–15.0 mm standard length [SL]), (2) juveniles from the previous lunar pulse (15–25 mm SL, although most were 20–25 mm SL), and (3) fish that were estimated to have settled more than one month previously (>25 mm SL). The distance from the sand base was measured with a tape measure, as was the distance to the top of the reef edge. The relative height above the bottom, as a percentage of the distance from the base, was then calculated for each individual. The substratum that they were closest to was also recorded, with the three most common being: rubble (broken coral that has lost structure and is largely eroded), dead coral (dead standing coral with some algal growth and invertebrates), live hard coral (mostly *Poc. damicornis*). To maintain independence of replicates, only the details of one randomly chosen individual fish was collected for each sampling point. Thirty random fish were chosen for each species-by-ontogenetic-stage combination. In addition, juveniles (>25 mm SL) of each species that did not have the other species within a 2 m radius were also sampled for their relative height and substratum association. Individuals that fitted this sampling constraint were harder to find in the study area, so replication was lower (n = 26 and 24, for *P. amboinensis* and *P. moluccensis*). A comparison of the distribution of these >25 mm SL juveniles when the two species were in close proximity to when they were not, yielded the potential influence of competition on the species distribution.

### Competitive Ability

#### Influence of species interactions on distribution

Newly metamorphosed juveniles of each fish species were placed on a patch reef composed of similar sized piece of dead, algae covered and healthy live *Poc. damicornis*, a common bushy hard coral. Dead coral was placed on the sand to form a base and a live colony of *Poc. damicornis* was placed on top to form a patch of ∼15×15×20 cm. This arrangement was how the habitats were commonly found. Patch reefs were at a distance of 5 m or more from the reef edge. To assess space use of solitary fish one light trap caught fish of each species was placed separately on a reef and their distribution and behaviour was quantified (see details below) after an acclimation period of 40 to 60 min (*P. amboinensis* n = 30, *P. moluccensis* n = 31). A pilot study had determined that there was no difference in space use whether an acclimation period of 40 min or 2 h were used, so a minimum period of 40 to 60 min was used for the main study (Fig. S2). To assess whether the presence of a potential competitor influenced their distribution and behaviour, a size matched pair of newly metamorphosed *P. amboinensis* and *P. moluccensis* (±0.2 mm) were placed on the patch reefs, and their distribution and behaviour recorded after 40 to 60 min (n = 21). A smaller study that left fishes on the reefs for 18–24 h found similar patterns of distribution to data collected after 60 min (Fig. S3). A fine mesh cage (6 mm mesh size; 30×30×30 cm dimensions) was placed over the top of the coral patches immediately after release of the fish to minimize the likelihood of predatory encounters and then carefully removed prior to assessment of behaviour. Similar numbers of the three treatments were conducted each day over a 5 d period on 20 individually labeled patch reefs with treatments allocated randomly to patch reefs. Fish were released between 10∶00 to 11∶30 h and behaviourally assessed between 11∶00 and 14∶00 h.

#### Body size and prior residency

The effects of body size and prior residency on behavioural interactions were examined for juvenile *P. amboinensis* and *P. moluccensis* in a crossed design: body size (3 levels: same size; *P. amboinensis* 3 mm SL larger than *P. moluccensis*; *P. moluccensis* 3 mm SL larger than *P. amboinensis*) and prior residency (2 levels: none; or 1 hour prior residency for *P. moluccensis*). Patch reefs composed of live and dead *Poc. damicornis* (as above) were established on sand away from the shallow reef edge. Either one individual of either species (the resident) was placed on a patch 60 min prior to the introduction of an individual of the second species, or both fishes were released onto the patch reef at the same time. Sixty minutes was found to be more than enough time for fish to explore the small patch reef and determine appropriate shelter sites. To reduce the potential confounding influence of individuals within the prior residency manipulation having been associated with small patch reef habitat for different amounts of time, the non-resident in the manipulation was placed on a similar patch reef 2 m from the experimental reef during the acclimation period of the resident, before its transfer to the resident’s reef. Fish were either the same size, ∼3 mm larger or ∼3 mm smaller than each other (mean SL and range: *P. amboinensis*, 25.0 mm, 20–29.5 mm; *P. moluccensis*, 24.7 mm, 20–29 mm). Juveniles used in the experiments were collected from the reef edge, and stored individually in 9 L plastic bags within a catch bag for 1–2 h prior to release onto an experimental patch reef. Size was measured through the plastic bag with calipers (±0.1 mm). Approximately 60 min after being paired with a heterospecific the behaviour and space use for both fishes was quantified (see below).

#### Behavioural observations

The behaviour of fish on the patch reefs was assessed over 3-min periods by a scuba diver positioned ∼1.5 m away from the patch using the protocol of McCormick (2009). A magnifying glass (4x) aided the assessment of bite rates and space use over the 3 min focal animal sampling period. Six aspects of activity and behaviour were assessed: a) total distance moved (estimated over the 3-min period); b) distance ventured from the coral patch (categorized as % of time spent within 0, 2, 5 or 10 cm away from the patch); c) height above substratum (categorized as % of the time spent within the bottom, middle or top third of the patch); d) boldness (recorded from observations over the whole 3 minutes as a continuous variable on a scale from 0 to 3 at 0.5 increments, where: 0 is hiding in hole and seldom emerging; 1 retreating to hole when scared and taking more than 5 sec to re-emerge, weakly or tentatively striking at food; 2 shying to shelter of patch when scared but quickly emerging, purposeful strikes at food; and 3, not hiding when scared, exploring around the coral patch, and striking aggressively at food). At the end of the 3 min observation period, the fish was approached with a pencil and the fish’s reaction and latency to emerge from shelter was taken into account in the assessment of boldness; e) number of fin displays; f) the number of chases or bites; g) number of avoidance episodes in response to a conspecific. Two additional variables were devised from these variables to summarise information and reduce the number of variables that were required in analyses. Relative height on the patch was summarized as a cumulative percentage of the time spent at varying heights over the 3 min observation period, with the top of the patch taken as height of 1, mid a height of 0.5, and bottom a height of 0. An aggression index was also created by adding the number of displays to the product of three times the number chases/bites and then subtracting the number of avoidance events. A weighting factor of 3 was used in conjunction with the chases/bites as the influence of this behaviour on the spatial distribution of the recipients appeared to be many times greater than their response to displays [31]. Dominant and subordinate individuals were decided based on the aggression index; dominant individuals had a positive score, whilst subordinates always had a negative score.

#### Growth comparison

Juvenile growth of both species was determined by the examination of the microstructure of cross sections of the sagittal otoliths. Collections of juvenile *P. moluccensis* were made where *P. amboinensis* was absent within a 1.5 m radius (and vice versa), and where the species was present. Fish were collected with a hand-net and an anesthetic clove oil/ethanol/seawater solution. Fish were euthanized with an overdose of clove oil, and then preserved in 70% ethanol prior to processing. Otoliths were processed according to the methods of [44].

#### Survival

*P. amboinensis* and *P. moluccensis* new recruits were placed onto patch reefs individually and paired in two size combinations to make five treatments (number of trials are in brackets): *P. amboinensis* (A) alone (n = 28); *P. moluccensis* (M) alone (n = 29); A < M (n = 20); M = A (n = 16); A > M (n = 18). The larger fish were ∼2 mm SL larger than the fish it was paired with. Fishes for this experiment were collected from light traps as before, kept in 30 L aquaria in the laboratory for one week supplied with aerated seawater and fed twice a day ad libitum with *Artemia* sp. nauplii. Keeping them for a week accentuated the size difference so that sufficient replicates of each size combination could be undertaken, but assured that all fishes were equally naïve to reef based predators and competitors. Patch reefs were established as before, but for the trials involving pairs of fishes, both fishes were released onto the patch reef at the same time. Reefs were then enclosed by a fine mesh cage for 40–60 min, and then removed and the presence of fishes monitored 2–3 times per day for 48 h. When a fish was missing it was assumed to have died. Our previous studies on newly settled damselfishes that have been tagged for individual recognition show that migration between patches is minimal or non-existent (e.g. [33], [40]).

### Analysis

#### Habitat use

The relative height of fish from the field sampling of spatial patterns was tested for equality across the three ontogenetic stages of juveniles sampled, and between the two species, using a two-factor ANOVA (i.e. Species and Ontogenetic stage). The nature of the significant interaction was further explored with Tukey’s HSD a posteriori tests. Using residual analysis, data was found to conform to the assumptions of normality and homogeneity of variance. The probability of *P. moluccensis* occurring on live coral was tested against the probability of *P. amboinensis* occurring on live coral using a binomial probability test.

A two-factor ANOVA was performed to determine whether the relative heights on the patch reef of *P. amboinensis* and *P. moluccensis* differed between species (factor: Species) or whether they were on their own (i.e. solitary) or together (factor: Context). Type III sums of squares were used since the design was unbalanced.

**Figure 1:**
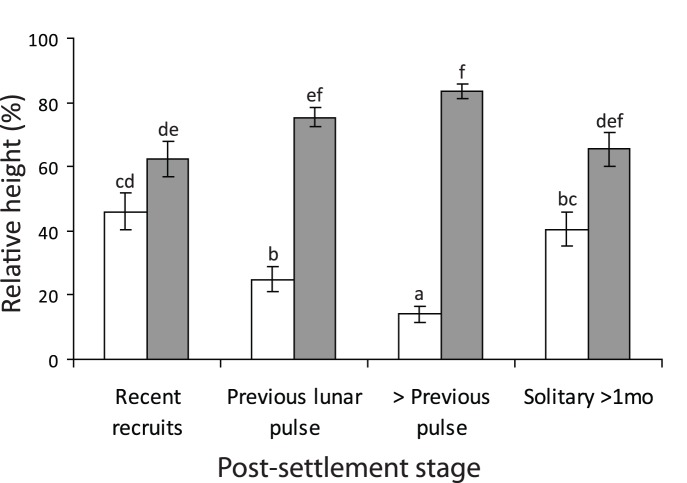
Vertical distribution of damselfish juveniles. The relative height above sand of three size groups of *Pomacentrus amboinensis* (white) and *P. moluccensis* (grey) juveniles on the reef edge, together with their height when they are not with the second species. Size or post-settlement age classes were: recent recruits (within the last week, ∼10–15.0 mm SL); juveniles from the previous lunar pulse (15–25 mm SL); juveniles that settled more than one month previously (>25 mm SL). Error bars are standard errors. Letters above bars represent Tukey’s HSD groupings. n = 30, except for the last two bars where n = 26 and 24.

#### Influence of species interactions on distribution

The prior residency versus size experiment was analysed to determine whether the height on the patch reef and behaviour of juvenile *P. moluccensis* changed in the presence of similar or different sized *P. amboinensis* using a repeated measures ANOVA. The difference in height on the patch of *P. moluccensis,* total distance moved and their boldness was compared 60 min after its introduction onto the patch reef, and then 60 min after the additional introduction to a patch reef of a *P. amboinensis* of one of three relative sizes (repeated measures factor: Time, alone versus together; Factor, Relative size). Because of the dominance of *P. amboinensis* in all situations except for when *P. moluccensis* was larger, trials that examined changes in space use and behavior of resident *P. amboinensis* before and after the introduction of a *P. moluccensis* were only conducted for the situations when *P. moluccensis* were larger than the resident *P. amboinensis*. Paired sample t-tests were used to test for differences in height, boldness and total distance (cm) moved in 3 min. Bonferonni correction was employed on these t-tests to account for 3 dependent tests (α′ = 0.017).

Log-linear modeling was used to examine the impact of body size and prior residency on dominance. Only size had a significant influence on the outcome of interactions regardless of the order in which terms were entered into the model, so interpretation was straightforward. Prior residency and body size were the explanatory variables with the number of wins of the different species the response variable. Winning was defined as when a fish was dominant, as measured by the aggression index (a combination of displays, chases, bites and avoidance events, as described above). A logistic regression was used to further demonstrate the role of body size and to predict the size difference needed to win a competitive outcome between the two species. The log-linear analysis and the logistic regression were preformed in the S-Plus for windows version 8.0 (S-Plus 2007).

**Figure 2:**
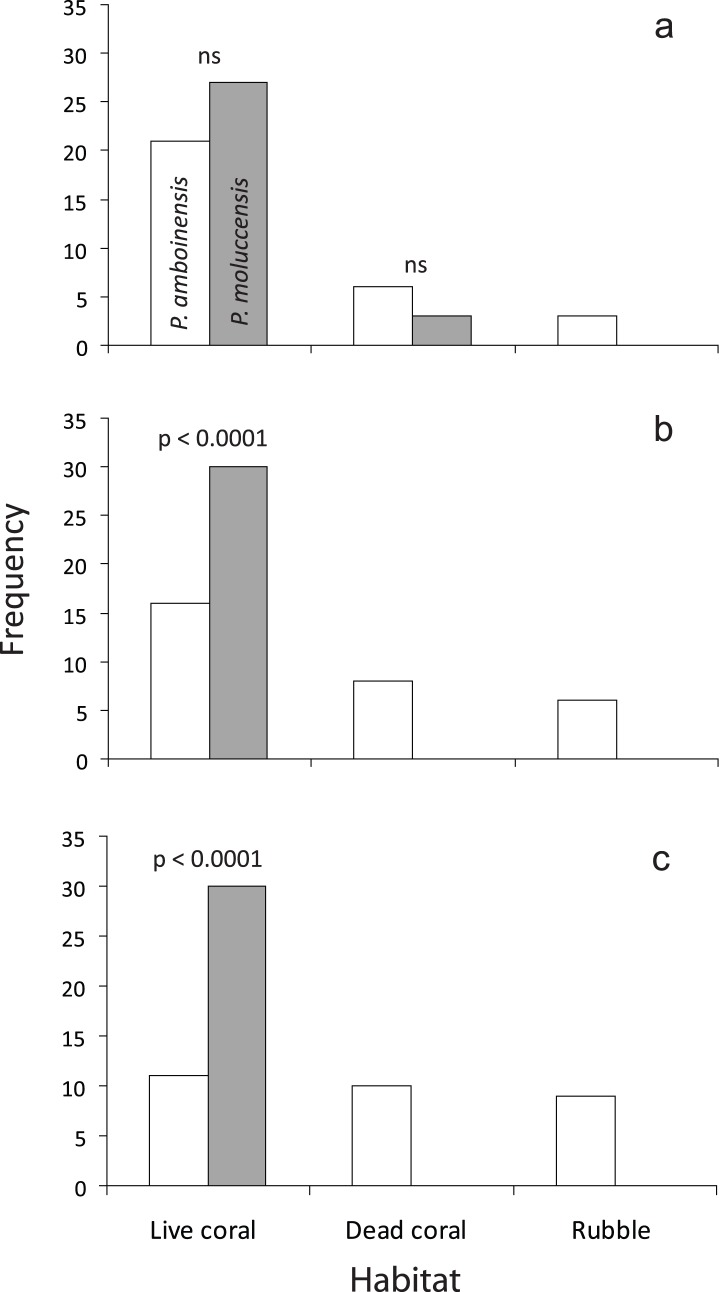
Use of the three main habitats at the reef edge by *Pomacentrus amboinensis* (white) and *P. moluccensis* (grey) of three different age groups of juveniles: a) recent recruits; b) 1 week to 1 month post-settlement; c) greater than 1 month. Results of binomial tests comparing the frequency of use of live coral of *P. moluccensis* to that of *P. amboinensis* are given.

#### Growth comparison

A two-factor repeated measures ANOVA (RMANOVA) was undertaken to compare otolith increment width trajectories between species and grouping (i.e., competitor present or absent). Multivariate tests were used for the repeated measures (within sample) component of the tests due to their robustness to violations of assumptions [45].

#### Survival

Multi-sample survival analyses using a Cox’s proportional hazard model compared the survival of fish in the 4 treatments through the 48 h census period for each species separately. For *P. amboinensis* there were 82 valid observations, involving 36 censored and 46 uncensored observations. Kaplan–Meier survival plots were used to illustrate mortality trajectories. Cox’s two-sample F-test were employed to test for differences in survival between pairs of treatments.

**Figure 3:**
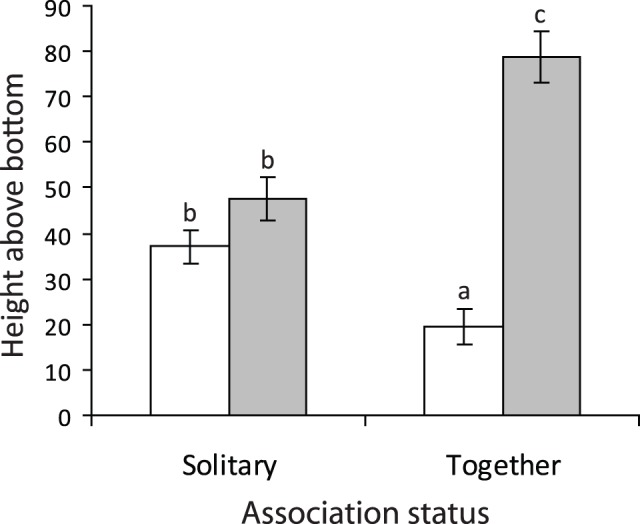
Relative height of *Pomacentrus amboinensis* (white) and *P. moluccensis* (grey) when solitary on a patch reef or when together. Error bars are standard errors. Letters above bars represent Tukey’s HSD groupings. Replicates per treatment: solitary *P. amboinensis* 30; solitary *P. moluccensis* 30; together 21.

## Results

### Habitat Use

The relative height of fish on the reef edge changed with ontogenetic stage but the trend differed between species (i.e. significant Species × Stage interaction: F_3,222_ = 16.813, p<0.0001). Recent recruits occurred, on average, midway up the reef edge regardless of species (Fig. 1). Within a couple of weeks the height on the reef diverged between species, with *P. moluccensis* frequenting the top 25% of the reef edge, while *P. amboinensis* occurred in the bottom 25% of the reef edge (Fig. 1). When juveniles from the previous pulse (>1 mo post-settlement) were sampled that did not have the other species nearby they tended to occur closer to the middle of the reef, which for *P. amboinensis* was significantly higher than juveniles that had *P. moluccensis* nearby (Fig. 1).

**Table 1:** Competitive outcome for *Pomacentrus amboinensis* from trials between juvenile *P. amboinensis* (A) and *P. moluccensis* (M) of various relative sizes on patch reefs composed of the bushy hard coral *Pocillopora damicornis*.

		Outcome for *P. amboinensis*	
Residency	Size	Win	Lose
No prior residency	A<M	1	9
	A = M	10	2
	A>M	12	0
*P. moluccensis* prior residency	A<M	1	10
	A = M	9	1
	A>M	10	0
*P. amboinensis* prior residency	A<M	0	10

Both *P. amboinensis* and *P. moluccensis* were associated with live *Pocillopora* with a similar high frequency as recent recruits (∼80%), and both also associated with dead coral to a lesser extent (15%). Only *P. amboinensis* was found close to rubble around settlement (Fig. 2a). After approximately one week, *P. moluccensis* was solely associated with live *Pocillopora*, while *P. amboinensis* had a significantly lower association with live *Pocillopora* (100% vs 53%, Fig. 2b) and also utilized dead coral and rubble. The patterns of habitat association for the older juvenile (>1 mo) *P. moluccensis* were similar to younger juveniles, while the patterns of habitat association for *P. amboinensis* was evenly distributed among the three habitat categories (Fig. 2c).

**Figure 4:**
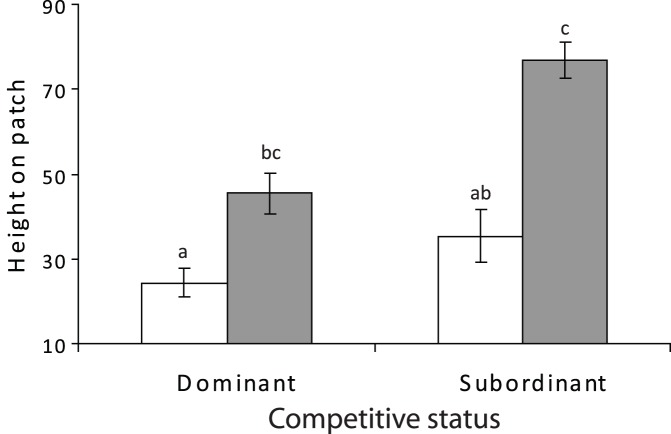
Influence of dominance status on distribution on patch reefs. Comparison of the relative height on the patch reef of *Pomacentrus amboinensis* (white) and *P. moluccensis* (grey) juveniles when they are dominant or subordinant whilst in a pair. The height index ranges between 0 (reef base) to 100 (reef top). n = 24, 10, 10, 24 (left to right). Error bars are standard errors.

### Competitive Ability

#### Influence of species interactions on distribution

Presence of the other species affected the height occupied on a habitat patch compared to when individuals were alone (Context × Species interaction: F_1,99_ = 28.20, p<0.001; Fig. 3). When alone on patch reefs, juveniles of both species occupied a similar position on the patch reefs, on average 45–55% from the base (Fig. 3). When similar sized fish were placed on patch reefs together, their distribution significantly changed, with *P. moluccensis* occupying the top of the patch and *P. amboinensis* the base (Fig. 3). With this partitioning of space came increases in displays, chases and avoidance behaviours, with *P. amboinensis* being the more dominant of the size-matched pair (Aggression index: F_1,40_ = 11.192, p = 0.0018; means: *P. amboinensis* 6.33, *P. moluccensis* −0.43).

**Figure 5:**
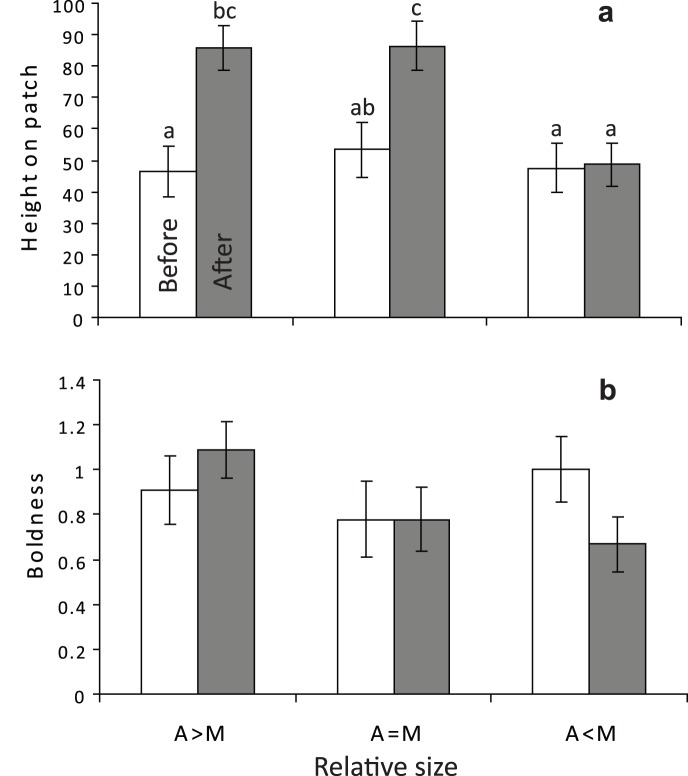
Influence of juvenile *Pomacentrus amboinensis* (A) of three relative sizes (greater equal or smaller) on the behavior of juvenile *P. moluccensis*. Data shows the percentage height above the bottom of the patch reef (a) and their boldness (0–3 index; b) of *P. moluccensis* after being alone on the patch reefs for 40–60 min (Before; white), and 40–60 min after (After; grey) the introduction of a *P. amboinensis* juvenile.

#### Body size and prior residency

Body size significantly affected competitive ability between *P. amboinensis* and *P. moluccensis* providing the only statistically significant response regardless of the order it entered into the model (Χ^2^ = 59.86, df 8, p<0.001). Prior residency of either species of damselfish did not influence the outcome of the interactions between species. The dominant species was usually determined by body size where the larger individual had a superior competitive ability (Table 1). However, when the species were of equal body size, there was a clear dominant species, with *P. amboinensis* winning the majority of the trials. This was further demonstrated with a logistical regression which was used to predict the proportion of *P. moluccensis* wins from the size difference between the two species (Fig. S4). This regression implies that not only is the outcome of competitive interactions with *P. amboinensis* based on body size but also on species. The fitted curve suggests that *P. amboinensis* was likely to win a competitive interaction even when it was up to ∼1.15 mm smaller than *P. moluccensis*.

**Figure 6:**
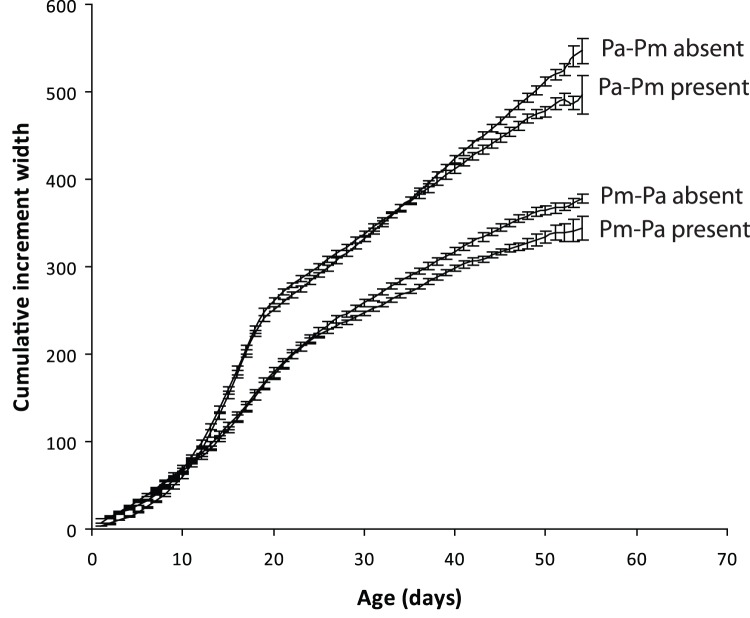
Growth in the field of *Pomacentrus amboinensis* (Pa) and *P. moluccensis* (Pm) in the presence or absence of eachother. Growth is expressed as otolith increment widths (µm). Means with standard errors are plotted (n = 25, 25, 30, 30 bottom to top, with variable n’s after age 45 d down to  = 4 minimum).

#### Competitive behavior

Height occupied on the patch reef was not affected by whether the fish was the dominant or subordinate for *P. amboinensis*, but dominance status did markedly influence the distribution of *P. moluccensis* (Species × Status: F_1, 64_ = 5.698, p = 0.01; Fig. 4). *P. amboinensis* consistently occupied the lowest parts of the patch reef. In contrast, *P. moluccensis* occupied the middle part of the reef when dominant, but occupied the highest parts of the reef when subordinate (Fig. 4).

Dominance status but not species influenced the maximum distance ventured in the 3 min observation period (Dominance status, F_1,64_ = 12.979, p = 0.0006; Species, F_1,64_ = 0.691, p = 0.409; Dominance × Species, F_1,64_ = 0.235, p = 0.629). Subordinates were three-times further from the reef than were dominants regardless of species (mean 0.6 cm versus 1.9 cm respectively). Neither dominance status nor species affected the total distance moved in 3 min (or the interaction; p>0.37 for all terms).

**Figure 7:**
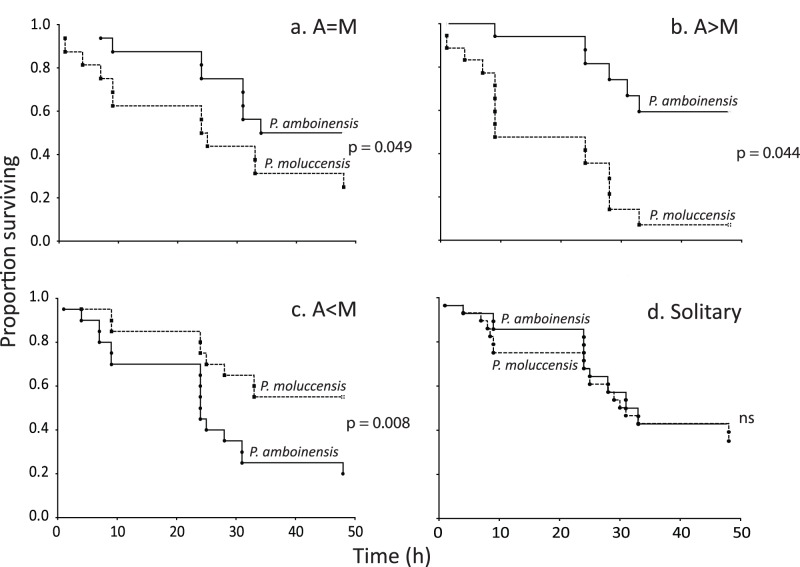
Comparison of juvenile fish survival in three different size combinations plus where they are solitary. a) *Pomacentrus amboinensis* (A) larger than *P. moluccensis* (M); b) *Pomacentrus amboinensis* same size as *P. moluccensis*; c) *Pomacentrus amboinensis* smaller than *P. moluccensis*; d) survival on patch reefs when alone. P-values are from tests between survival trajectories displayed in each graph by Cox two-sample F-tests (ns, non-significant).

Species identity influenced bite rate, with newly settled *P. amboinensis* having a lower bite rate than *P. moluccensis* recruits (4.9 versus 7.9 bites per min respectively; F_1,64_ = 4.100, p = 0.047). There was also a trend for dominance status to affect feeding rate, but this was not significant at α = 0.05 (dominant 4.9, subordinate 6.9 bites per min; F_1,64_ = 3.715, p = 0.058) and there was no interaction between Species and Dominance status. There was no statistically significant relationship between fish standard length and bite rate for either species regardless of whether they were subordinate or dominant (r <0.03, p>0.05).

#### Impact of a competitor on space use

The height occupied by juvenile *P. moluccensis* on experimental patch reefs depended on the size of *P. amboinensis* that was also present on the patch (Fig. 5a). When the *P. amboinensis* placed onto the reef was larger or the same size as the *P. moluccensis* then the *P. moluccensis* moved toward the top of the patch (Fig. 6a). When *P. amboinensis* was smaller than *P. moluccensis* both species positioned themselves on average at a similar height on the coral patch (Fig. 5a). This change in distribution of *P. moluccensis* with the size of the interacting *P. amboinensis* yield a significant Time by Size interaction in the repeated measures ANOVA (F_2,29_ = 7.690, p = 0.002). The boldness of *P. moluccensis* was also found to change depending upon the relative size of the interacting *P. amboinensis* (Fig. 5b; F_2,29_ = 4.746, p = 0.016), though Tukey’s HSD post-hoc tests did not yield significant results at α = 0.05. The addition of *P. moluccensis* that was larger than the resident *P. amboinensis* led to a doubling of the total distance moved of the *P. amboinensis* (alone, 3.6 cm, together 6.9 cm; Paired t-test, t_9_ = 2.95, p<0.016), but no change in height on the reef or boldness (paired t-tests, NS at α = 0.017).

#### Growth comparison

Otolith growth differed between species (RMANOVA Time × Species: Pillai’s Trace_48,36_ = 0.966, p<0.0001), with each species showing a significant change in growth through time and with social grouping (competitor present or absent) (Time × Grouping: Pillai’s Trace_48,36_ = 0.723, p = 0.019). These trends through time were similar between species (TimexGroupingxSpecies: Pillai’s Trace_48,36_ = 0.525, p = 0.729), with higher otolith growth being displayed when the competitor was absent (Fig. 6). Differences in growth appeared to be initiated rapidly after settlement.

#### Survival

There was an overall difference between the four treatments in the survival of *P. amboinensis* when placed on patch reefs with varying sizes of *P. moluccensis* or on their own (χ_3, 0.05_ = 8.79, p = 0.032). Kaplan-Meier plots suggested that *P. amboinensis* survived worst when it was on patches with a larger *P. moluccensis*, and best when it was on a reef where it was paired with a smaller *P. moluccensis* (Fig. S5a). *P. amboinensis* had significantly higher survival when paired with a smaller *P. moluccensis* than when alone (Cox-F two-sample test F_12,32_ = 2.122, p = 0.044). Likewise, the survival of *P. moluccensis* juveniles differed depending upon whether they were placed on a patch reef with a *P. amboinensis* that was larger or smaller than themselves (χ_3, 0.05_ = 10.28, p = 0.016). *P. moluccensis* survived worst when it was on patches with a larger *P. amboinensis*, and best when it was on a reef where it was paired with a smaller *P. amboinensis* (Fig. S5b). In contrast to *P. amboinensis*, there was no significant difference in survival of *P. moluccensis* when paired with a smaller *P. amboinensis* compared to when alone (Cox-F two-sample test F_18,36_ = 1.583, p = 0.118).

When *P. amboinensis* was on patch reefs with similar sized *P. moluccensis* they survived better than *P. moluccensis* (Cox F_16,24_ = 2.089, p = 0.049; Fig. 7a). *P. amboinensis* survived best when it was larger than *P. moluccensis* (Cox F_12,30_ = 4.654, p = 0.0003; Fig. 7b). Likewise, *P. moluccensis* survived best when placed on patch reefs with a smaller sized *P. amboinensis* (Cox F_18,32_ = 2.629, p = 0.008; Fig. 7c). In contrast, the mortality did not differ between the species when they were alone on the patch reefs (Cox F_32,36_ = 1.133, p = 0.357; Fig. 7d).

## Discussion

To predict how communities will respond to changes in the biotic environment, ecologists must evaluate the strength of interactions among species and their consequences for community structure and dynamics. However, this information is only useful if we understand the caveats under which one species will prosper at the expense of the other. The present study suggests that interspecific competition within a cohort has a marked influence on the distribution and survival of fishes around settlement. It is expected that interspecific interactions among fishes within a guild will be most intense when individuals are of similar size [46]. Evidence suggests that while the two species of damselfish prefer the same settlement habitat, the overlap in distribution rapidly diminishes over the first month on the reef. Our experiments suggested that this was partly due to strong interspecific competition between juveniles, which had a negative impact on growth and survival of the subordinate species. While in general there was a negative effect of interspecific interactions, having the subordinate species in close proximity enhanced the short-term survival of the dominant species in the days following settlement when mortality was highest. The study highlights the importance of intracohort interactions between species immediately after settlement in influencing small scale community dynamics.

Low risk shelter appears to be the focus of the competition between the two species examined. Generally, competition significantly increases the chance of predation by displacement of subordinate, weaker competitors to riskier locations (e.g. [31], [47], [48]) or the dominant aggressively acquiring higher quality living space from subordinate individuals [46], [49], [50]. Dominant individuals in the current study spent the majority of their time at lower levels on the patch reef where there were more shelter holes compared to subordinate individuals. Attempts by the smaller or subordinate fish to occupy the lower reaches of the coral patch were met with displays, chases and bites from the dominant regardless of species. It is likely that ‘enemy-free space’ is a limiting resource competed for by most small organisms in areas where predation is high (e.g. [32]).

The present study found evidence of a trade-off between exposure to predators and best access to planktonic food resources. Being high up and further out from shelter should be advantageous for obtaining primary access to food particles carried on water currents. Tropical waters are typically characterized as food-limited, and studies that enhance food lead to elevated growth rates [12]. When competitors were of the same or smaller size, *P. amboinensis* was the dominant competitor and interactions with dominant individuals pushed *P. moluccensis* higher up the coral patch. For both species, survival of recently settled juveniles was lowest when their interspecific competitor was slightly larger. Previous studies of *P. amboinensis* that have explored the intraspecific interactions of recently settled juveniles have shown that dominant individuals also stay closer to the bottom of the reef and venture less far from shelter [31], [51]. Subordinates, which are always smaller than dominant individuals in *P. amboinensis*, are pushed higher up and further away from the reef where they are more vulnerable to predators and exhibit higher levels of mortality [31]. Interestingly however, other studies have shown that larger, dominant individuals typically avail themselves of the highest quality food resources (e.g. [52], [53]). For damselfishes there is a growth advantage to getting access to planktonic food first as they are highly selective feeders. Surprisingly, in the present study there was a trend for subordinates to have higher bite rates than dominant fish regardless of species. There was no evidence that this higher intake was to fuel greater activity levels as the total distance moved did not differ with either dominance status or species. Subordinates therefore appear to have first access to food brought to their habitat patch by currents and also a higher feeding rate, which should sum to a higher calorific intake, and possibly higher short term growth. At least in the short term, there was a trade-off to having greater access to food higher up and further away from the patch at the juvenile life-stage because this subordinate position makes them more accessible to predators. Dominant individuals chose the conservative and safer option of being close to shelter, but in doing so they have poorer access to food.

Ontogenetic stage may account for the differences in the relationship between consumption and dominance status among studies. We examined newly settled fishes, while other studies that have quantified this relationship have examined older life stages. Meekan et al. [51] found that there was a change in the relationship between size, boldness and foraging rate for *P. amboinensis* when the behaviour of newly-settled fish was compared to individuals from the same cohort one month later. McCormick & Meekan [51] argued that strong selection pressure during the first few days after settlement may promote behavioural flexibility around settlement. Recent research has found that most marine and freshwater fishes have relatively poor innate recognition of predators ([54]–[56] for exceptions), but a single concordance of damage-released olfactory cues with either the smell or sight of an organism will label that organism as a potential threat [57]. Diet cues from predators [57] and repetitive exposure of threat cues with cues from a particular organism reinforce some species as being of greater threat than others [58]. This information can be quickly passed between newly-settled individuals through social learning [59], [60]. The end result is that, while newly-settled fish are highly vulnerable to predators, they quickly learn to respond appropriately to potentially threatening species and become less vulnerable, which in part explains their rapid increase in survival probability in the days following settlement [61]. For the damselfishes studied, there is a covariance between aggression and propensity to take risk at this early settlement stage with the most aggressive individuals positioning themselves closest to shelter. The subordinates appear to either be forced, or to actively adopt, a diametrically opposed behavioural mode of being in a high risk position. Here, away from shelter, they can gain greater access to food and achieve a higher feeding rate. While individuals that adopt this latter strategy are exposed to a higher probability of death, they may achieve a higher growth rate which may have an advantage later in life because survival is often correlated with growth on broader temporal scales [62]. Temporally, dominant fishes therefore appear to initially trade-off higher growth for the safety of shelter, and once they better understand the risks associated with their new environment they adopt less risk-adverse use of space that promotes high growth.

The presence of an interspecific competitor of a slightly smaller size enhanced the survival of the dominant species over that found when the species was alone on a similar habitat patch. This enhanced survival was most marked in the more dominant species, *Pomacentrus amboinensis*. Most other studies document either no effect on survival of the dominant species, or reductions in survival of the subordinate or both species in the presence of an interspecific competitor (e.g. [11], [48], [63]). Behavioural observations of interactions between the species in the current study suggest that the survival benefit is not due to *P. amboinensis* adopting more risk adverse behaviour in the presence of the competitor. While the subordinate *P. moluccensis* was forced further up and off the reef edge by aggression from *P. amboinensis*, the latter stayed close to the reef base. Increased survival may also have been a product of increased vigilance in the presence of a subordinate. There was a strong trend in the present study for dominant individuals to have a lower foraging rate and this may be because fish have shifted their attention to the activities of the subordinate and protection of their shelter. Many studies have found that foraging is one of the most sensitive indicators of increased vigilance (e.g. [58], [64]).

The outcome of interactions between these two species of damselfish was based on body size of individuals and was asymmetrical. There was a size-related threshold for dominance; juvenile *P. amboinensis* had to be 1.2 mm shorter than *P. moluccensis* before it became subordinate. Since body size is directly related to age of individual fish it is possible that an individual’s competitive aptitude is a learned ability that increases with age of the fish. Asymmetrical interactions of this type are common among fishes and other organisms [65] and have been shown both intra-specifically (e.g. [6], [9], [47], [49], [53], [66]–[72]) and inter-specifically (e.g. [4], [11], [46], [48], [50], [73]–[80]). In the present study, *P. amboinensis* was found to be dominant once we had accounted for size. Robertson [46] suggested that it is possible that a species effect could counter the effects size has on dominance capabilities among territorial reef fish; however, it has rarely been demonstrated (see [78]). Our study demonstrates that species effects can supplant a modest but ecologically relevant size effect between species within a cohort.

Prior residency of the coral patch did not affect the outcome of interactions in these two species of damselfish. Though prior residency was found to be a strong factor in previous studies (e.g. [23], [49], [50], [78]), placing a resident on the reef an hour prior to the other competitor did not provide smaller individuals of either species with a competitive advantage, or affect the species dominance of *P. amboinensis* when individuals were of similar size. This lack of a prior residency advantage may be because of the relatively short time residents had to familiarize themselves with their habitat patches. It is expected that the intensity of prior residency effects will be related to the time available for resource preemption [81] and while these damselfish very rapidly establish themselves on habitat patches (because failure means death) habituation for longer time periods are likely to give rise to stronger resource defence [23]. Few studies address the mechanisms underlying priority effects and it is unclear the extent to which these simply represent advantages driven by a covariance between the time established and its size or age, experience or exploitative competition through resource depletion. All three have previously been shown to affect competitive superiority [10], [82], [83]. Additional experiments are required to tease apart the relative effects of size and priority over longer time frames.

The present study found that interspecific interference competition within cohorts of settling fishes was important in influencing the distribution and survival of individuals and was mediated through aggressive interactions. Intraspecific competition has often been shown to have similar or greater effects on life history characteristics and distributions than interspecific competition [84]. Indeed, previous studies on our dominant species, *Pomacentrus amboinensis*, have shown a similar magnitude of effect immediately upon settlement [31]. Individuals rapidly establish size-based dominance hierarchies [31], [51] and it is the personality of individuals exhibited immediately after settlement that, in part, determines who survives the highly selective mortality during this ecological and physiological transition period [33]. In this species, intraspecific interactions between adults and juveniles indirectly influence the abundance patterns and also the distribution of life history traits within a population, through their influence on selective mortality [42]. This intraspecific effect is habitat-related and operates through juveniles surviving better in male nesting territories, which are constructed in rubble patches at the base of the reef. All these interactions occur within the first month after settlement when it is estimated that 90% of settling individuals can be removed from the population (e.g. [61]). These studies highlight the key importance of focusing on the juvenile fish and their behavioural interactions within and among species, in the context of the environment in which they live, if we are to better understand the processes that influence the distribution of site attached organisms on reefs.

## Supporting Information

Figure S1 Reef fish community on the reef edge of shallow reef at Lizard Island, on the northern Great Barrier Reef. The yellow fishes are *Pomacentrus amboinensis* and *P. moluccensis*, the focus of the present study.
(DOC)

Figure S2 Comparison of pilot study results based on a 2 h acclimation prior to behavioural assessment, and results from a 40–60 min acclimation.
(DOC)

Figure S3 Comparison of pilot study results based on an 18–24 h acclimation prior to behavioural assessment, and results from a 40–60 min acclimation when pairs of fish are placed on isolated patch reefs.
(DOC)

Figure S4 Results of a logistic regression examining the relationship between the proportion of wins and the size difference between two species of damselfish juveniles, *Pomacentrus amboinensis* and *P. moluccensis*.
(DOC)

Figure S5 Survival curves of newly settled (a) *Pomacentrus amboinensis* (‘A’) and (b) *P. moluccensis* (‘M’) on isolated *Pocillopora* hard coral reefs on their own (solitary) and paired with the other species of a similar or different size (2 mm size difference).
(DOC)

Text S1 Gut contents analysis of *Pomacentrus amboinensis* and *P. moluccensis*
(DOC)
