# Supplementary material for: It Pays to Be Pushy: Intracohort Interference Competition between Two Reef Fishes
Source: PLoS One. 2012 Aug 10;7(8):e42590. doi: 10.1371/journal.pone.0042590 (PMC3416846; doi:10.1371/journal.pone.0042590)
Supplement: Figure S1 — Reef fish community on the reef edge of shallow reef at Lizard Island, on the northern Great Barrier Reef. The yellow fishes are Pomacentrus amboinensis and P. moluccensis, the focus of the present study. (DOC) [file pone.0042590.s001.doc]

**
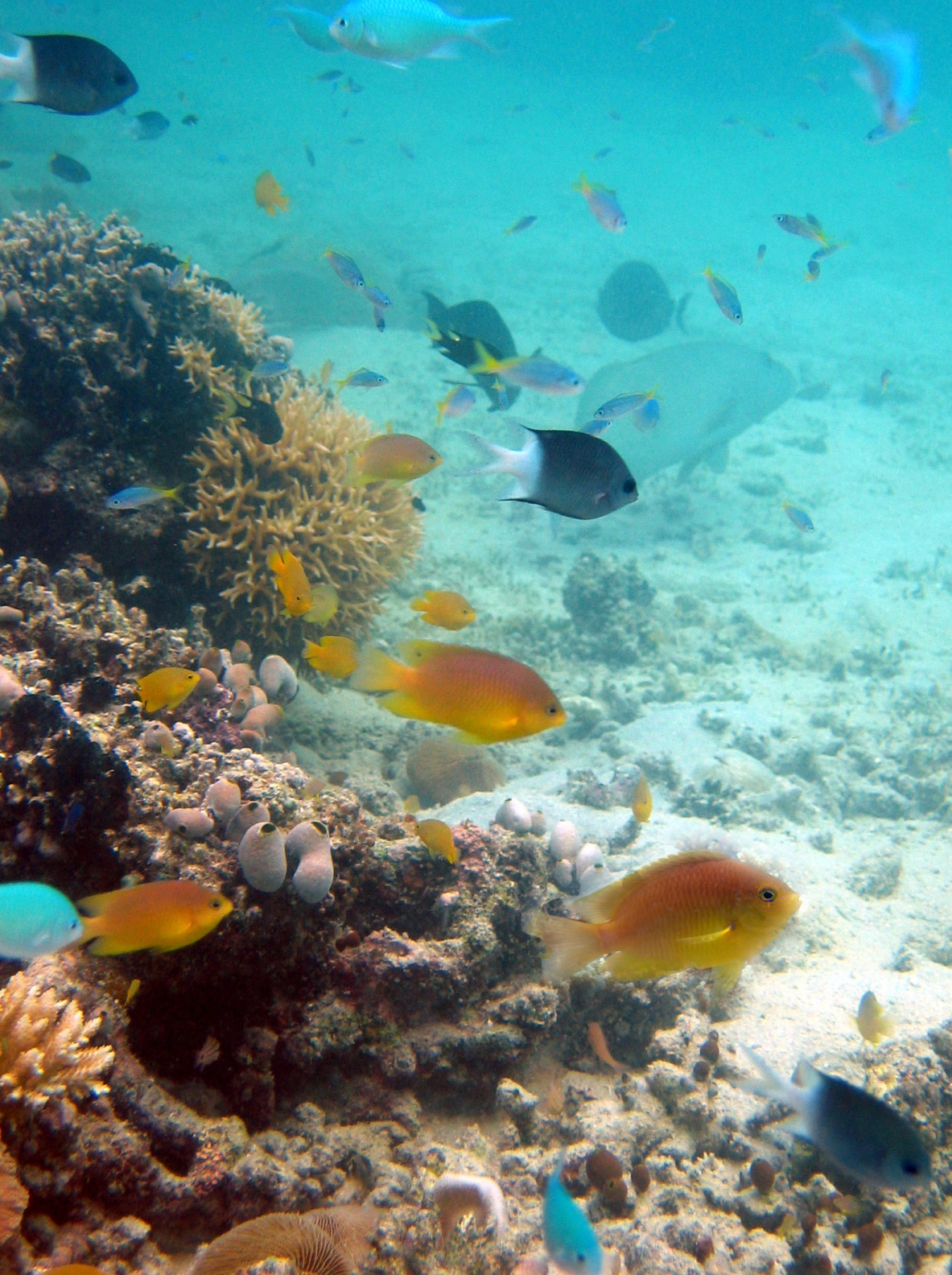
**

**Figure S1.** Reef fish community on the reef edge of shallow reef at Lizard Island, on the northern Great Barrier Reef. The yellow fishes are *Pomacentrus amboinensis* and *P. moluccensis*, the focus of the present study.
