# Supplementary material for: It Pays to Be Pushy: Intracohort Interference Competition between Two Reef Fishes
Source: PLoS One. 2012 Aug 10;7(8):e42590. doi: 10.1371/journal.pone.0042590 (PMC3416846; doi:10.1371/journal.pone.0042590)
Supplement: Figure S2 — Comparison of pilot study results based on a 2 h acclimation prior to behavioural assessment, and results from a 40–60 min acclimation. (DOC) [file pone.0042590.s002.doc]

**Figure S2.** **Comparison of pilot study results based on a 2h acclimation prior to behavioural assessment, and results from a 40-60min acclimation.** Displayed are the mean relative heights of *Pomacentrus amboinensis* (white) and *P. moluccensis* (grey) when solitary on a patch reef (± SE). Replicates per treatment: 40-60min *P. amboinensis* 30, *P. moluccensis* 30; 120min *P. amboinensis* 8, *P. moluccensis* 7. Neither acclimation time nor species affected the height above substratum (Acclimation F_1,72_ = 0.931, p = 0.338; Species F_1,72_ = 2.80, p = 0.098; Acclimation x Species F_1,72_ = 0.0001, p = 0.991).
