# Supplementary material for: It Pays to Be Pushy: Intracohort Interference Competition between Two Reef Fishes
Source: PLoS One. 2012 Aug 10;7(8):e42590. doi: 10.1371/journal.pone.0042590 (PMC3416846; doi:10.1371/journal.pone.0042590)
Supplement: Figure S3 — Comparison of pilot study results based on an 18–24 h acclimation prior to behavioural assessment, and results from a 40–60 min acclimation when pairs of fish are placed on isolated patch reefs. (DOC) [file pone.0042590.s003.doc]

**Figure S3. Comparison of pilot study results based on an 18-24h acclimation prior to behavioural assessment, and results from a 40-60min acclimation when pairs of fish are placed on isolated patch reefs.** Displayed are the mean relative heights of *Pomacentrus amboinensis* (white) and *P. moluccensis* (grey) (± SE). Replicates per treatment: 40-60min *P. amboinensis* 21, *P. moluccensis* 21; 18-24h *P. amboinensis* 5, *P. moluccensis* 5. Neither Acclimation time nor the interaction with Species significantly affected height above the bottom (Acclimation, F_1,48_ = 0.243, p = 0.624; Acclimation x Species, F_1,48_ = 1.113, p = 0.297). Height was affected by Species (F_1,48_ = 78.172, p < 0.0001).
