# Supplementary material for: It Pays to Be Pushy: Intracohort Interference Competition between Two Reef Fishes
Source: PLoS One. 2012 Aug 10;7(8):e42590. doi: 10.1371/journal.pone.0042590 (PMC3416846; doi:10.1371/journal.pone.0042590)
Supplement: Figure S4 — Results of a logistic regression examining the relationship between the proportion of wins and the size difference between two species of damselfish juveniles, Pomacentrus amboinensis and P. moluccensis. (DOC) [file pone.0042590.s004.doc]

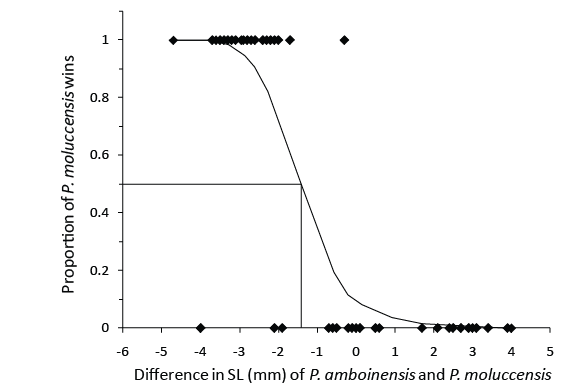


**Figure S4. Results of a logistic regression examining the relationship between the proportion of wins and the size difference between two species of damselfish juveniles, *Pomacentrus amboinensis* and *P. moluccensis*.** Winning was defined as when a fish was dominant, as measured by the aggression index. The fitted curve suggests that *P. amboinensis* becomes more likely to win when its size difference is 1.15mm smaller than *P. moluccensis*.
