# Supplementary material for: It Pays to Be Pushy: Intracohort Interference Competition between Two Reef Fishes
Source: PLoS One. 2012 Aug 10;7(8):e42590. doi: 10.1371/journal.pone.0042590 (PMC3416846; doi:10.1371/journal.pone.0042590)
Supplement: Figure S5 — Survival curves of newly settled (a) Pomacentrus amboinensis (‘A’) and (b) P. moluccensis (‘M’) on isolated Pocillopora hard coral reefs on their own (solitary) and paired with the other species of a similar or different size (2 mm size difference). (DOC) [file pone.0042590.s005.doc]

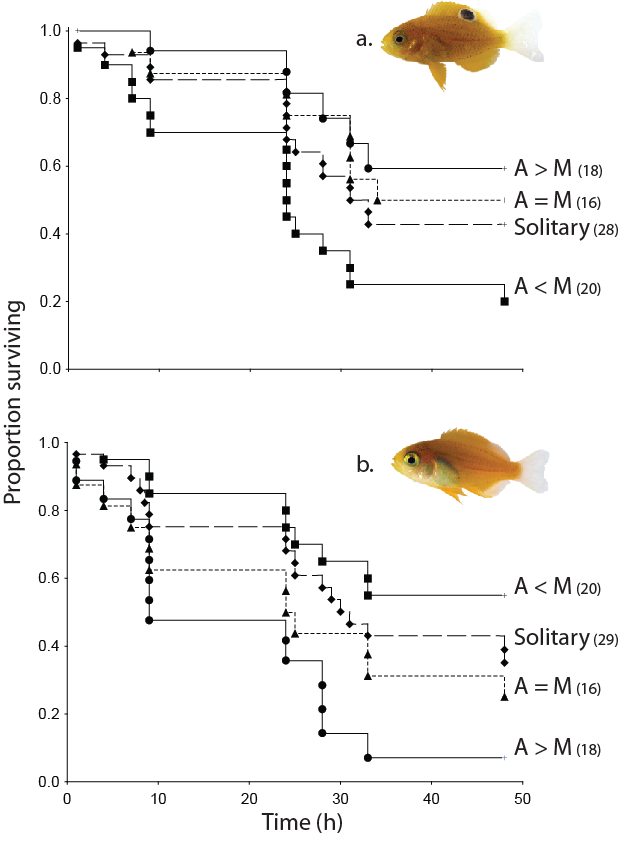


**Figure S5. Survival curves of newly settled (a) *Pomacentrus amboinensis* (‘A’) and (b) *P. moluccensis* (‘M’) on isolated *Pocillopora* hard coral reefs on their own (solitary) and paired with the other species of a similar or different size (2 mm size difference).** Graphs are Kaplan-Meier survival plots with the number of replicate trials in brackets.
