# Supplementary material for: It Pays to Be Pushy: Intracohort Interference Competition between Two Reef Fishes
Source: PLoS One. 2012 Aug 10;7(8):e42590. doi: 10.1371/journal.pone.0042590 (PMC3416846; doi:10.1371/journal.pone.0042590)
Supplement: Text S1 — Gut contents analysis of Pomacentrus amboinensis and P. moluccensis (DOC) [file pone.0042590.s006.doc]

**Supplement text 1. Gut contents analysis of *Pomacentrus amboinensis* and *P. moluccensis***

*Gut contents*

Fish were anesthetized with clove oil and caught with hand nets. Fish were taken every twenty minutes from the water and placed on ice in order to slow prey digestion (Kent et al. 2006). The fish were returned to the station where their stomach cavities were injected with 4% phosphate buffered formaldehyde in order to preserve the stomach content and were stored in vials of the same liquid until the time of dissections. Fish were weighed and measured (standard length, SL). The contents of the stomach were removed and the type of prey under 25 randomly located dots was identified in order to estimate the percent volume of each prey type in broad categories. A MANOVA that included the four main food categories found that the species did not differ in the contents (Pillai’s Trace _4,45_ = 0.02, p = 0.920). On average their stomach’s contained: 4.4% cladocera, 4.1% calanoid copepods, 9.1% harpacticoid copepods, 3.1% mollusc and 79.3% unidentifiable matter. Correlations found there was no statistically significant correlations between size of individuals and the amount of a certain prey item consumed for either species.
